# Supplementary material for: The Distribution and Predictive Factor of Extra-Pancreatic Malignancy Occurrence in Patients with Pancreatic Intraductal Papillary Mucinous Neoplasm—A Ten-Year Follow-Up Case–Control Study in Taiwan
Source: Cancers (Basel). 2024 Dec 7;16(23):4102. doi: 10.3390/cancers16234102 (PMC11640256; doi:10.3390/cancers16234102)
Supplement: Supplementary file 1 [file cancers-16-04102-s001.zip › cancers-3291892-supplementary.pdf]

Supplement Table S1. Indication for the original imaging which picked up the pancreas cyst

CASE1	abdominal pain
CASE2	abdominal pain
CASE3	CA-199 elevated
CASE4	Bone fracture accidentally found
CASE5	cancer followed accidentally found
CASE6	CA-199 elevated
CASE7	abdominal pain
CASE8	pancreatitis
CASE9	abdominal pain
CASE10	abdominal pain
CASE11	cancer followed accidentally found
CASE12	cancer followed accidentally found
CASE13	pancreatitis
CASE14	CEA elevated
CASE15	CA-199 elevated
CASE16	abdominal pain
CASE17	health examination
CASE18	abdominal pain
CASE19	CEA elevated
CASE20	abdominal pain
CASE21	body weight loss
CASE22	pancreatitis
CASE23	pancreatitis
CASE24	cancer followed accidentally found
CASE25	AFP elevated
CASE26	abdominal pain
CASE27	abdominal pain
CASE28	abdominal pain
CASE29	abdominal pain
CASE30	health examination
CASE31	abdominal pain
CASE32	abdominal pain
CASE33	health examination
CASE34	cancer followed accidentally found
CASE35	HCV f/u echo accidentally found

CASE36	abdominal pain
CASE37	body weight loss
CASE38	cancer followed accidentally found
CASE39	abdominal pain
CASE40	abdominal pain
CASE41	pancreatitis
CASE42	abdominal pain
CASE43	jaundice
CASE44	jaundice
CASE45	cancer followed accidentally found
CASE46	abdominal pain
CASE47	abdominal pain
CASE48	pancreatitis
CASE49	abdominal pain
CASE50	cancer followed accidentally found
CASE51	abdominal pain
CASE52	abdominal pain
CASE53	abdominal pain
CASE54	abdominal pain
CASE55	cancer followed accidentally found
CASE56	abdominal pain
CASE57	abdominal pain
CASE58	abdominal pain
CASE59	health examination
CASE60	health examination
CASE61	abdominal pain
CASE62	abdominal pain
CASE63	pancreatitis
CASE64	pancreatitis
CASE65	jaundice
CASE66	pancreatitis
CASE67	abdominal pain
CASE68	pancreatitis
CASE69	abdominal pain
CASE70	health examination
CASE71	pancreatitis
CASE72	abdominal pain

CASE73	body weight loss
CASE74	abdominal pain
CASE75	abdominal pain
CASE76	abdominal pain
CASE77	abdominal pain
CASE78	health examination
CASE79	abdominal pain
CASE80	abdominal pain
CASE81	body weight loss
CASE82	elevated CEA
CASE83	cancer followed accidentally found
CASE84	abdominal pain
CASE85	cancer followed accidentally found
CASE86	health examination
CASE87	abdominal pain
CASE88	abdominal pain
CASE89	cancer followed accidentally found
CASE90	cancer followed accidentally found
CASE91	cancer followed accidentally found
CASE92	abdominal pain
CASE93	cancer followed accidentally found
CASE94	cancer followed accidentally found
CASE95	cancer followed accidentally found
CASE96	abdominal pain
CASE97	abdominal pain
CASE98	cancer followed accidentally found
CASE99	abdominal pain
CASE100	abdominal pain
CASE101	abdominal pain
CASE102	health examination
CASE103	health examination
CASE104	abdominal pain
CASE105	abdominal pain
CASE106	cancer followed accidentally found
CASE107	abdominal pain
CASE108	abdominal pain
CASE109	health examination

CASE110	CEA elevated
CASE111	abdominal pain
CASE112	cancer followed accidentally found
CASE113	abdominal pain
CASE114	health examination

Supplement Table S2. Comparison of incidence between incidence of cancers in Taiwan and incidence of EPM after IPMN diagnosed

	Incidence of cancers in Taiwan	Incidence of EPM after IPMN diagnosed
Extra-pancreatic malignancy (total)		
Colon cancer	$89.6/10^5$(0.09%)	3/114(2.6%)
Lung cancer	$72.9/10^5$(0.07%)	8/114(7.0%)
Hepatocellular carcinoma	$46.7/10^5$(0.04%)	5/114(4.3%)
Urothelial cancer	$7.3/10^5$(0.007%)	3/114(2.6%)
Breast cancer	$128.3/10^5$(0.13%)	1/114(0.8%)
Ovarian cancer	$15.3/10^5$(0.01%)	1/114(0.8%)
Head and neck cancer	$35.7/10^5$(0.03%)	1/114(0.8%)
Skin cancer	$16.6/10^5$(0.02%)	2/114(1.7%)
Kaposi’s sarcoma	$3.9/10^5$(0.004%)	1/114(0.8%)
Lymphoma	$9.3/10^5$(0.009%)	1/114(0.8%)
